# The contributions of normal variation and genetic background to mammalian gene expression

**DOI:** 10.1186/gb-2006-7-3-r26

**Published:** 2006-03-31

**Authors:** Colin Pritchard, David Coil, Sarah Hawley, Li Hsu, Peter S Nelson

**Affiliations:** 1 Divisions of Human Biology, Fred Hutchinson Cancer Research Center, Seattle, WA, 98109, USA; 2 Clinical Research, Fred Hutchinson Cancer Research Center, Seattle, WA, 98109, USA; 3 Public Health Sciences, Fred Hutchinson Cancer Research Center, Seattle, WA, 98109, USA

## Abstract

**Background:**

Qualitative and quantitative variability in gene expression represents the substrate for external conditions to exert selective pressures for natural selection. Current technologies allow for some forms of genetic variation, such as DNA mutations and polymorphisms, to be determined accurately on a comprehensive scale. Other components of variability, such as stochastic events in cellular transcriptional and translational processes, are less well characterized. Although potentially important, the relative contributions of genomic versus epigenetic and stochastic factors to variation in gene expression have not been quantified in mammalian species.

**Results:**

In this study we compared microarray-based measures of hepatic transcript abundance levels within and between five different strains of *Mus musculus*. Within each strain 23% to 44% of all genes exhibited statistically significant differences in expression between genetically identical individuals (positive false discovery rate of 10%). Genes functionally associated with cell growth, cytokine activity, amine metabolism, and ubiquitination were enriched in this group. Genetic divergence between individuals of different strains also contributed to transcript abundance level differences, but to a lesser extent than intra-strain variation, with approximately 3% of all genes exhibiting inter-strain expression differences.

**Conclusion:**

These results indicate that although DNA sequence fixes boundaries for gene expression variability, there remain considerable latitudes of expression within these genome-defined limits that have the potential to influence phenotypes. The extent of normal or expected natural variability in gene expression may provide an additional level of phenotypic opportunity for natural selection.

## Abstract 2

Analysis of microarray-based transcript levels within and between five different mouse strains show that 23-44% of all genes exhibit differences in expression levels between genetically identical individuals.

## Background

Biological entities such as individual cells, organs, and entire organisms display phenotypes that are simultaneously dictated and constrained by the composition of nucleic acids comprising their genomes. Differences in DNA sequence between individuals within the same species may produce qualitative and quantitative alterations in gene expression that influence biochemical processes conferring disease susceptibility and the beneficial or adverse responses to pharmacological intervention [1,2]. Thus, a critical component of biomedicine centers on establishing the cause, extent and result of gene expression variability with an aim toward establishing pathological associations. To this end, the development of technologies such as DNA microarrays have allowed for quantitative assessments of transcriptional activity for thousands of genes simultaneously [3]. Microarray-based methods have been used to measure transcriptional variance in a variety of organisms, including yeast [4], flies [5], fish [6], mice [7], and men [8]; usually in the context of assessing the contribution of gene expression to phenotypic attributes of age, sex, strain, or disease. While the major component of phenotypic diversity within species is thought to be provided by combinations of heritable variations in DNA, it is readily apparent that individuals sharing nearly identical genomes, such as inbred mouse strains and monozygotic twins, may exhibit strikingly different characteristics [9,10].

To assess the extent and nature of gene expression variability both within populations of genetically identical individuals and between genetically heterogeneous individuals, we selected five strains of commonly used laboratory mice; inbred *129*, *Balb/c*, and *FVB*, and outbred *CD1 *and *CFW*, isolated RNA from the livers of three males from each strain, and quantified transcript abundance levels by comparative hybridizations to cDNA microarrays.

## Results

Mice bred for more than 60 generations should fix the vast majority (potentially all) of genetic contribution to variation [11], and thus individual mice within each inbred strain are considered genetically identical. We studied the liver in view of its important contribution to a wide variety of metabolic processes as well as practical considerations involving sample quantities and the ease of tissue procurement. To account for technical inconsistencies and facilitate comparisons within and between strains, each array hybridization used a common reference consisting of RNA combined from the liver, testes, and kidney of all mice used in the experiments. Two replicate arrays were performed for each individual mouse liver sample with each of two different fluorescent dyes to control for potential dye bias, thereby generating 4 replicate arrays per mouse and a total of 60 arrays.

We anticipated that three major sources of measurable variation in transcript levels would be represented in this dataset. The first involves the technical inconsistencies in experimental procedures and was assessed by the four replicate arrays performed for each mouse sample. The second source of variation is represented predominantly by intrinsic and extrinsic non-genetic factors influencing gene expression. This variance component was measured through the determination of transcript levels between mice of the same strain with identical genomes. All mice were matched for age, and were provided consistent diets and living environments. The third source of gene expression variability was expected to be driven by differences in DNA sequence or genome structure between the different mouse strains. This inter-strain variability was measured by determining transcript abundance levels between mice of different strains.

To identify genes whose transcript levels varied between genetically identical individuals, we first used an ANOVA model with a conservative assessment of significance [7]. This method yielded the following number of variable genes within each strain: *129*, 37 genes; *Balb/c*, 36 genes; *CD1*, 26 genes; *FVB*, 21 genes; and *CFW*, 11 genes. Our previous study of liver gene expression in *C57BL/6 *mice identified 21 variable genes (0.8% of all genes assessed), indicating that the overall experimental results are quite consistent [7]. While this method identifies variable genes with high confidence, we concluded that the approach has a high rate of false negatives and is unduly restrictive when one is interested in assessing overall levels of variability rather than focusing on any particular gene product.

We next employed a less conservative strategy that involves controlling the positive false discovery rate (pFDR) [12]. We chose a level of acceptable false positives of 10% such that among the identified variable genes, about 10% probably do not actually vary. Separate analyses within each strain identified 554 (23%) genes exhibiting variability among individual *129 *mice, 1,059 (44%) genes among *Balb/c *mice, 749 (31%) genes among *CD1 *mice, 610 (26%) genes among *FVB *mice, and 661 (28%) genes among the *CFW *mice (Table 1). In a joint analysis in which all strains were evaluated simultaneously, 1,876 genes (79%) varied within strain at a pFDR of 10% (see Materials and methods). Overall, mice in the *Balb/c *strain exhibited greater liver gene expression variability than mice from other strains. For a gene to be identified as variable, either the transcript level difference between individual mice is large, or the array variance - in this case the technical variability - is small. We specifically re-evaluated the array variability and did not identify a lower array variance in the *Balb/c *experiments.

Of the genes exhibiting variable expression levels within strains, 33 were variable within all 5 strains, and 154 were variable in 4 of the 5 strains (see Additional data file 2, supplemental Table 2). To determine how many genes are expected to be in common by chance if genes were chosen randomly, we undertook a simulation study with 50,000 datasets generated by randomly selecting groups of 554, 1,059, 749, 610, and 661 genes from the 5 strains for each data set and determined the number of genes represented in all 5 sets. The greatest number of genes in common, by chance alone, was 19, though typically fewer than 10 genes were found in common. This analysis indicates that the 33 genes identified in our study represent a highly significant level of overlap (*p* < 0.00002). Searches based on gene ontology (GO) classification indicated that genes associated with cell growth [GO:0008151], cytokine activity [GO:0005125], amine metabolism [GO:0009308], and the ubiquitin ligase complex [GO:0000151] were enriched among the genes with consistent intra-strain variation, when compared to the array as a whole. All genes showing significant inter-individual variability in our previous study of *C57BL/6 *mice [7] also varied in at least one strain analyzed in the current study. Moreover, genes previously found to exhibit substantial hepatic intra-strain variability, including *CisH*, *Hhex*, C*yp4a14*, and *Gadd45a*, varied in at least three out of the five strains. Interestingly, four genes identified in the current study are involved in the ubiquitination process; *Wsb1*, *Arih1*, *Cdc27*, and *Chordc1*. This finding suggests the possibility that normal variability in protein degradation pathways could provide an additional level of global gene expression variability either through direct targeting of specific proteins or via a cascade of indirect effects influencing transcriptional regulation.

**Figure 1:**
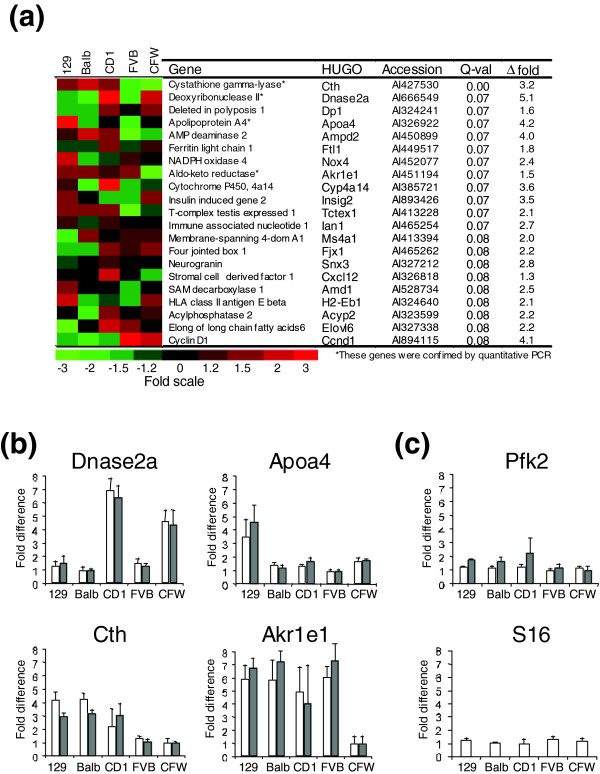
Genes exhibiting inter-strain expression variability. **(a) **Genes with the most statistically significant inter-strain variance are shown using a color scale to indicate relative expression levels in the five strains. ESTs and uncharacterized transcripts are not shown. Red indicates higher expression and green indicates lower expression relative to other strains. The q-values (Q-Val) indicate the probability that each gene is falsely discovered as variable between strains. Δ fold refers to the difference in gene expression levels between the strains with the highest and lowest measurements. **(b) **Confirmation of transcripts with variable expression. Quantitative RT-PCR measurements of transcripts encoding apolipoprotein A-IV (*ApoA4*), *Dnase2*, aldo-keto reductase (*Akr1e1*), and cystathione gamma-lyase (*Cth*). Open bars represent results of RT-PCR quantification. Gray bars represent results of microarray quantification. **(c) **Confirmation of transcripts with stable expression. Quantitative RT-PCR measurements of transcripts encoding phosphofructo-kinase 2 (Pfk2), and ribosomal protein S16. S16 expression levels were used to normalize real-time PCR data, although there was not more than a 1.5-fold difference in S16 expression between any two mice. Results are expressed as fold differences relative to the lowest expressing strain for each gene (set to a value of 1). Error bars indicate the standard deviation of 12 microarray or 9 real-time PCR experiments.

We next sought to assess the gene expression variability between different mouse strains. For this analysis we used an ANOVA model in which we considered the F ratio of mouse (between strain) to mouse (within strain) effect, where the significance of the F statistic is determined again by the pFDR (see Materials and methods). Using a pFDR of 10%, the analyses of individual mice identified 66 transcripts out of 2,382 (2.8%) that exhibited greater inter- than intra-strain variability (Figure 1a). Several transcripts exhibited substantial inter-strain variability, and we confirmed the microarray results for four of these genes by quantitative PCR (Figure 1b). Apolipoprotein A-IV (*ApoA4*) message levels varied 5.7-fold between mice of the *129 *strain and mice of the *FVB *strain, a finding that confirms previous studies demonstrating a high level of expression variability for this gene [13,14]. *ApoA4 *is synthesized in the liver and intestine, and is a mediator of plasma lipid transport. Human studies have identified polymorphisms in the *ApoA4 *gene that associate with *ApoA4 *plasma levels, inter-individual variability in cholesterol levels, and risk of coronary heart disease [15]. The regulation of *ApoA4 *expression involves both transcriptional and post-transcriptional processes influenced by genetic variation in the gene itself [14]. Other genes exhibiting high inter-strain differences in expression levels encode proteins modulating cellular oxidative stress responses. These include NADPH oxidase 4 (*Nox4*), cytochrome p450 4a14 (*CYP4a14*), glutathione S transferase pi (*Gstp*), peroxiredoxin 4, and ferritin light chain (*Ftnl*) (Figure 1a). Our results are consistent with previous studies that have demonstrated substantial mouse strain differences in basal iron status, ferritin levels and the potential for modulating oxidative hepatic stress. Immunoquantitation of total liver ferritin levels in four mouse strains determined a three- to fourfold difference between the *SWR *and *C57BL/6 *strains, with *Balb/c *and *DBA/2 *strains having levels between these extremes [16]. Our results identified the highest levels of *Ftl *expression in *CD1 *mice, a strain not examined in the previous report. This study and our previous work each determined that stress-response genes exhibit substantial individual or within-strain variability [7]. Thus, these inter-strain measurements represent a level of consistency superimposed on the underlying gene expression variability. The specific reason for the high representation of the stress-response genes in these experiments has not been determined. A rapid physiological response to the process of CO_2_-induced death could be contributory. If so, then these results indicate a robust strain-dependent physiological difference in the response to sacrifice. Alternatively, these gene expression patterns might reflect fundamental differences between strains relating to the generation and control of oxidative stress that could correlate with differences in lifespan and disease susceptibility.

**Figure 2:**
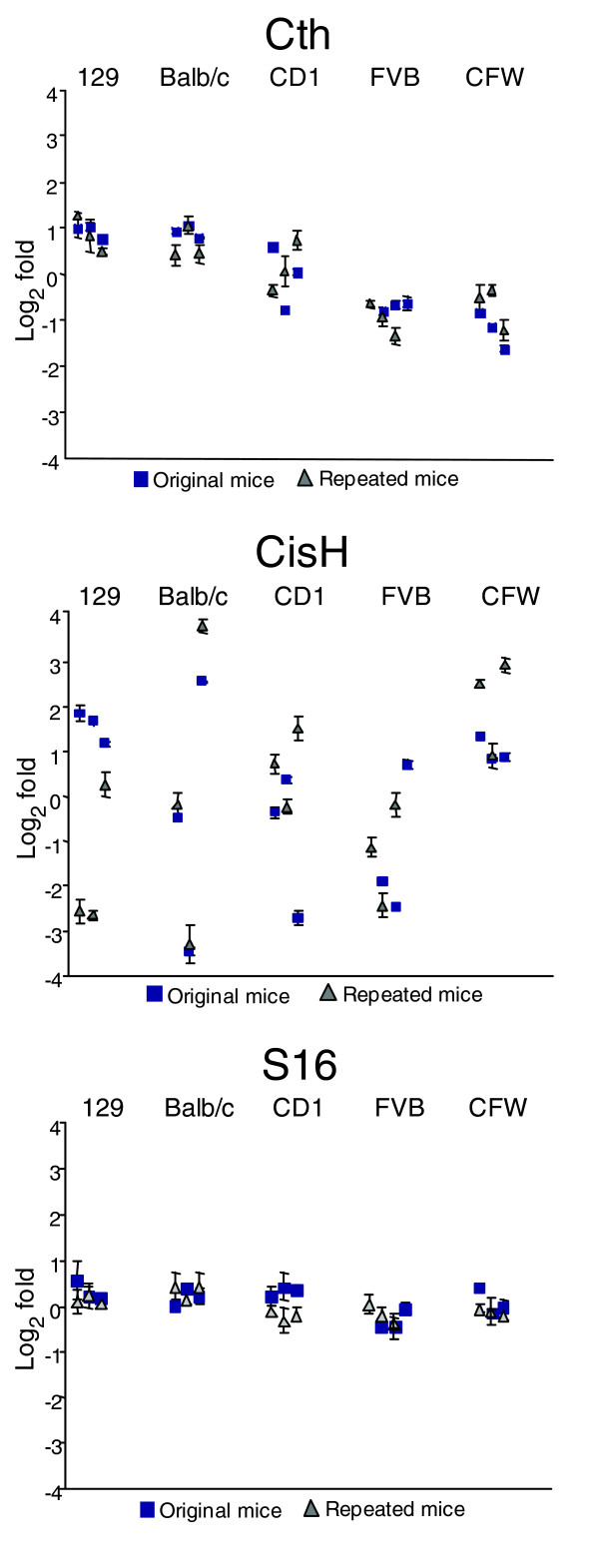
Reproducibility of variant and invariant gene expression characteristics. Quantitative RT-PCR measurements of *Cth*, *CisH*, and *S16 *transcript levels are shown for the original three mice used in the microarray experiments (blue squares), and a second cohort of three additional mice from each strain (gray triangles). The original mouse measurements for *Cth *and *S16 *are the same as in Figure 1c, but presented here in log_2 _scale. Mice in the second cohort underwent 4 independent RNA preparations from each liver (total of 12 RNA preparations per strain). Error bars represent the standard deviation of transcript measurements from the four RNA preparations, or from four replicate PCR reactions in the case of the original mice (note that for some measurements, the error bars fall within the square). Quantitative RT-PCR measurements of *Apoa4*, *Dnase2a*, and *Socs2 *from the additional mice are shown in Additional data file 2 (supplemental Figure 1).

To determine if the intra- and inter-strain gene expression measurements were reproducible, we examined selected genes in 3 additional mice from each strain, all males between 68 and 72 days old. To ensure that inconsistencies in RNA isolation and cDNA synthesis procedures were not contributing to variance, we resected the livers from the additional mice, divided each liver into four sections, and separately isolated RNA from each portion. We used quantitative RT-PCR to measure transcript levels for three genes that varied between strains but were stable within strain (*Cth*, *ApoA4*, *Dnase2a*), two genes that varied within strain (*Cish *and *Socs2*), and one gene that was stable both within and between strains (*S16*). The results demonstrated highly reproducible intra-individual measurements in the RNA samples isolated from the same mouse (standard deviation ± 0.4-fold), a result that indicates minimal technical variation associated with RNA preparations (Figure 2). Concordant with the results from the original mice, *Cth *exhibited a low level of intra-strain variability, but was expressed approximately fourfold lower in the *FVB *and *CFW *strains (Figure 2a). Measurements of *ApoA4 *and *Dnase2a *expression were also highly concordant with the original results (see Additional data file 2, supplemental Figure 1a,b). *CisH *and *Socs2 *expression again exhibited substantial within-strain variability, with measurements differing by up to 50-fold between *Balb/c *mice (Figure 2b; Additional data file 2, supplemental Figure 1c), while *S16 *expression remained quite stable across individuals and strains (Figure 2c).

**Figure 3:**
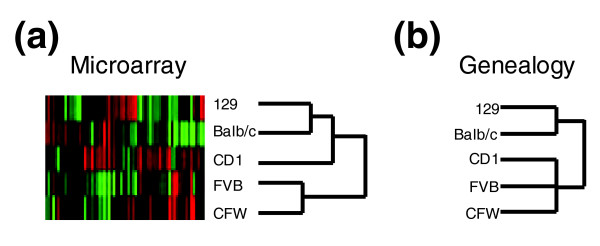
Gene expression and mouse strain relationships. **(a) **Mouse strain relationships based upon a hierarchical cluster analysis of the 66 genes exhibiting differential expression between strains with a pFDR of <10% (individual mouse analysis). **(b) **Mouse strain relationships based upon published genealogy [17].

We anticipated that transcripts expressed differentially between mouse strains would primarily reflect heritable differences in strain genomes. As such, mice sharing a common ancestry might be expected to exhibit similar variability in gene expression compared to more distantly related mice. Supporting this concept are studies in humans showing less variability in lymphocyte transcript levels between identical twins relative to siblings, and siblings relative to unrelated individuals [8]. We performed hierarchical clustering on the subset of genes exhibiting significant inter-strain differences and arranged the different mouse strains according to their similarities in expression profiles (Figure 3). Clustering based on the entire list of genes, either unweighted or weighted by F-values, produced similar results (data not shown). Comparing this expression-based dendrogram with the known phylogenetic relationship of these strains supports a genetic basis for a component of the expression variability [17].

**Figure 4:**
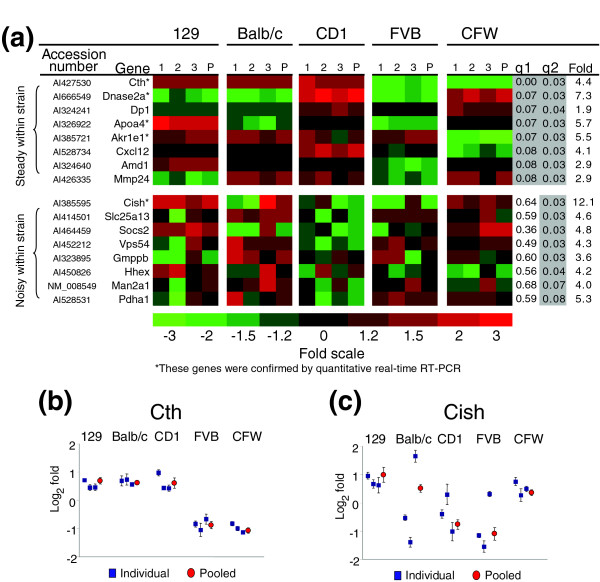
Comparison of transcript levels exhibiting inter-strain variability determined by analyses of individual samples and pooled samples. **(a) **Examples of genes that demonstrated significant inter-strain variance after pooling the RNA from three mice of each strain. The relative expression values are shown for the three mice individually (1, 2, 3) and for the three mice pooled (P). Note that the individual and pooled results are data from independent hybridizations. Q-values are listed for both the individual mouse experiment (q1) and the pooled mouse experiment (q2), and values less than 0.1 are shaded gray to indicate statistical significance. Genes with low intra-strain variability (stable within strain) were statistically significant in both the individual and pooled experiments, while genes that had large intra-strain variability (noisy within strain) were significant only in the pooled experiment. Asterisks denote genes that were verified by quantitative PCR. **(b) **Genes such as *Cth *were relatively stable within strain. **(c) **Genes such as *CisH *were relatively noisy within strain. Error bars indicate standard deviations of four microarray experiments.

To assess the effects of pooling on the ability to characterize inter-strain variation in liver gene expression, we performed a separate microarray analysis using RNA samples combined from the three mice of each strain. Four replicate arrays were hybridized for each of the five pooled strain samples with two replicates in each dye orientation. We identified 374 genes (about 15% of all genes included in the analysis) that varied significantly between strains using a pFDR of 10%. Of the 66 genes exhibiting significant inter-strain variability determined from analyses of individual mice, 60 were evaluable in this experiment and 41 (68%) were also determined to be variable in the analysis of pooled samples. These results indicate that a large portion of genes presumed to be variable between mouse strains and representing potential genetic determinants of quantitative phenotypic traits are actually quite noisy among individuals. This conclusion is easily visualized by plotting the transcript levels for individual mice and for pooled mice (Figure 4a). Genes, such as Cystathione gamma-lyase (*Cth*), that vary significantly in both the individual and pooled analyses showed relatively steady expression within each strain (Figure 4b), while genes that vary significantly only in the pooled analysis, such as *CisH*, tend to have high intra-strain variance (Figure 4c). This result emphasizes that for many genes the intra-strain or within-genotype variation is large, and a single pool of a small number of mice will not accurately reflect the population mean for the most variable genes.

## Discussion

Comprehensive studies of gene expression in model organisms such as *Saccharomyces *and *Drosophila *have delineated the contributions of age, sex, and genotype to corresponding variations in transcript levels. However, the size constraints of these species necessitates the use of sample pools composed of hundreds to millions of discrete organisms, an approach that eliminates the ability to assess variability at the level of the individual. In contrast, assessing the relationships between the genome and gene expression variability in humans is hampered by the inability to precisely control the multitude of environmental influences that profoundly influence gene expression in qualitative and quantitative ways. In this context, the mouse represents a useful model system highly suited for establishing that component of variability that is independent of diversity directly encoded in the genome. Measurements of intra-strain gene expression levels reflect the allowable latitudes of gene expression in any single individual in a fixed environment at a given point in time. The inter-strain measurements reflect the additional contribution of heterogeneity at the level of the genome.

Based on the analyses of transcript levels in individual mice, we found the greatest contribution to overall gene expression variability occurred among genetically identical individuals: 23% to 44% of all genes exhibited measurable variation, depending on strain (see Additional data file 2, supplemental Figure 3). Substantially less variance was attributable to genome differences between strains (about 2.8%). Few studies assessing natural gene expression variability in mammalian species that might provide a context for these findings have been reported. Analyses of transcript levels in skeletal muscle between five mouse strains found greater inter-strain than intra-strain differences. [18]. This suggests that muscle tissue exhibits a narrow range of normal variation relative to liver. However, the study design in which two mice per strain and two microarrays per mouse were compared provides substantially less statistical power to detect differences within strain. Interestingly, concordant with the findings reported here, Balb/c mice demonstrated the greatest level of intra-strain variation. A comparative analysis of mRNA abundance levels in the hippocampus of mice from 8 mouse strains identified more than 200 genes with significant strain differences using very stringent statistical criteria [19]. The experimental design involved tissue pooled from six mice of each strain, rather than individual mice. This pooling strategy was apparently based in part on the results of a prior microarray study indicating that transcript levels of genes expressed in the hippocampus of genetically identical mice were quite similar with only about 0.1% of all transcripts called differentially expressed [20]. It is possible that there is lower inter-individual variability in hippocampus than in liver. However, this previous study directly compared only pairs of mice in a head-to-head fashion, and the criteria for differential expression were based on a 1.7-fold change in abundance level, and not on statistical criteria.

Overall, we found that the expression of most hepatic genes in mice housed in standard 'steady-state' laboratory vivarium conditions is similar between individuals of the same or different strain. However, the transcript levels of a sizeable minority varied substantially. The proportion of genes exhibiting significantly variable expression between individual fish (18%) [6], yeast strains (24%) [4], and fly genotypes (25%) [5] is similar to that observed here between individual mice (23% to 44%). Analyses of gene expression in human tissues have also shown considerable variability between individuals. Importantly, substantial contributions to this variation cannot be attributed to genotypic differences between subjects [8,21,22]. Comparisons of transcript and protein levels between humans and non-human primates identified significantly greater variation among the human subjects than between humans and chimpanzees [23], a finding further supporting the conclusion that a sizeable component of transcript abundance measurements reflects non-genomic variation.

There are several possible contributors to the gene expression variability observed in genetically identical individuals. Technical factors include subclinical disease states, unrecognized differences in environments and diet, or heterogeneity in the cell-type compositions of the analyzed tissues. We attempted to precisely control environmental and handling effects during the design of this study, and we did not observe any histological differences in the cellular composition of livers within or between strains. The ideal experiment would assess temporal variation in tissue transcript levels within an individual mouse, but in the case of liver gene expression these measurements would be confounded by changes resulting from repeated tissue biopsies. Importantly, our analyses of separate liver samples acquired from the same mouse yielded highly concordant transcript measurements.

One component of inter-individual variability could be represented by stochastic events or noise. Recently, gene expression measurements at the level of the single cell have provided direct experimental evidence of quantifiable contributions of stochastic biochemical noise to phenotypic variation in isogenic populations [24,25]. The end-result of this component of variability has long been appreciated through studies of developmental processes that revealed requirements for feed-back amplifications of initial asymmetrical noise for cell fate determination [26].

A second potential contributor to individual differences in gene expression centers on epigenetic regulation. Methylation of cytosine residues in the CpG islands of gene promoters and the covalent modifications of histones represent two important epigenetic modifications that influence gene transcription. Recent studies emphasize the importance of these regulatory mechanisms for dictating phenotypes in individuals with minimal divergence in genome sequence. A provocative report by Rakyan *et al. *[27] determined that the penetrance of the highly variable kinky-tail phenotype found in the well-studied *Axin-fused *(*Axin*^*Fu*^) mouse strain correlated with the differential methylation of a retrotransposon within *Axin*^*Fu*^. Importantly, the methylation state of the retrotransposon was inherited transgenerationally after both maternal and paternal transmission, and was influenced by strain background. Striking differences in DNA methylation and histone acetylation have been observed in identical twins with increasing 'epigenetic drift' associated with advanced age [28]. Similar age-related epigenetic shifts have been reported in mice [29]. In the studies reported here, we found several genes that exhibited high variability in more than one strain, suggesting that certain genomic loci may be prone to imprecise regulatory control.

## Conclusion

In the context of complex multicellular organisms, the end-result of phenotypic diversity in the setting of a fixed genome has long been appreciated. Toxicology studies have repeatedly shown differing susceptibilities to drug effects, such as carcinogen-induced tumor promotion within isogenic mouse strains [30]. Genetically identical animals aged under tightly controlled environments exhibit wide ranges in lifespans [31]. Indeed, the seeming incongruity between genetic homogeneity and phenotypic variability was recognized more than 40 years ago [32]. Importantly, the magnitude of gene expression variability measured in this study suggests either a tolerance for wide abundance ranges of certain transcripts, or potentially an organismal advantage for maintaining a state of gene expression variability offering an additional level of phenotypic opportunity for natural selection.

## Materials and methods

### Animal work and RNA preparation

Mice were purchased from Charles River Laboratories (Wilmington, MA, USA), maintained in a barrier facility and cared for in accordance with an approved Animal Care and Use Committee (IACUC) protocol. All mice were between 68 and 73 days old and were housed in identical environments with the same diet (Harlan Teklad 8664), constant temperature (20 to 22°C), and consistent light and dark cycles (controlled photoperiod of 12 hour light/12 hour dark). Water was provided *ad libitum*. Three male mice were sacrificed from each of the following strains (nomenclature in italics is used throughout this paper): 129S4 (*129*), *Balb/c*AnNCrlBR (*Balb/c*), Crl:CD-1^®^(ICR)BR (*CD1*), *FVB*/NCrlBR (*FVB*), and Crl:CFW^®^(*SW*)BR (*CFW*); *CFW *is sometimes referred to as 'Swiss Webster'. Each mouse was brought individually into a separate room for sacrifice and killed in a CO_2 _chamber. The liver, left kidney, and left testis were removed from each mouse and immediately snap-frozen in liquid nitrogen. Care was taken to ensure that the minimum amount of time elapsed from the sacrifice of the first mouse to the last. Total RNA was extracted from the tissue using the TRIzol reagent (Life Technologies, Grand Island, NY, USA) according to the manufacturer's protocol. For an RNA reference standard, equal quantities of total RNA were combined from all three organs of all the mice. This same reference RNA was used on every array to standardize comparisons between arrays. For confirmation studies, 3 additional male mice of each strain, ages 68 to 72 days, were processed in a similar manner except that the liver was divided into 4 sections before snap-freezing.

### Microarray construction, probe generation, and data collection

Each microarray comprised 5,285 mouse cDNAs obtained from the Research Genetics' sequence-verified set of IMAGE clones (Research Genetics, Invitrogen Corporation, Carlsbad, CA, USA). All cDNA clones used for array construction were sequence verified and annotated accordingly. Clone inserts were amplified by PCR, purified, verified by gel electrophoresis and spotted onto polylysine-coated glass microscope slides using a GeneMachines (San Carlos, CA, USA) robotic spotter as described previously [7]. cDNA probes were generated from 50 μg of total RNA in a reaction volume of 30 μl containing oligo(dT) primer/0.2 mM amino acid-dUTP (Sigma-Aldrich, St Louis, MO, USA)/0.3 mM dTTP/0.5 mM each dATP, dGTP, and dCTP/380 units of Superscript II reverse transcriptase (Life Technologies). The purified cDNA was combined with either Cy3 or Cy5 monoreactive fluorophores (GE Healthcare, Piscataway, NJ, USA (formerly Amersham Pharmacia)) that covalently couple to the cDNA-incorporated aminoallyl linker in the presence of 50 mM NaHCO_3 _(pH 9.0). The experimental and reference probes were combined and competitively hybridized to microarrays under a coverslip in a volume of 24 μl for 16 h at 63°C. Slides were washed in graded sodium chrolide/sodium citrate buffer (SSC, 1× SSC = 0.15 M NaCl/0.015 M sodium citrate, pH 7) and spun dry. Array images were collected for the Cy3 and Cy5 emissions using a GenePix 4000A fluorescent scanner (Axon Instruments, Foster City, CA, USA). The image data were extracted and analyzed using GENEPIX 3.0 microarray analysis software (Axon Instruments).

### Data analysis

For each array spot, the intensity levels of the two fluorophores were obtained by subtracting median background intensity from median foreground intensity. A gene was only considered expressed if the fluorescence intensity of the corresponding spot was at least six foreground pixels greater than four standard deviations above background on every array. For each gene, the logarithm base 2 ratios (referred to henceforth as log ratios) of the two channels were calculated to quantify to relative expression levels between the experimental and reference samples. To allow for inter-array comparisons, each array was normalized to remove systemic sources of variation. This normalization was accomplished by means of a print-tip-specific intensity-based normalization method. [33]. A scatter-plot smoother, which uses robust locally linear fits, was applied to capture the dependence of the log ratios on overall log-spot intensities. The log ratios were normalized by subtracting the fitted values based on the print-tip-specific scatter-plot smoother from the log ratios of experimental and control channels. Examination of the spread of the normalized log ratios via boxplots indicated no systemic variation due to any experimental variable such as different batches of arrays or RNA preparations. Therefore, no scale adjustment was performed on the arrays before combining data across samples.

The expression of genes that vary among mice within each strain was evaluated using an ANOVA model (Pritchard *et al*. [7]). Here, an F-value with degrees of freedom 2 and 8 was used to assess the variability of mouse variance within each strain.

To identify genes that varied among strains of mice, a nested mixed effects ANOVA model was used. Specifically, the model is written as:

*y *= overall mean + dye + strain + mouse within strain

where y is the normalized log_2 _ratio and the mouse within strain is a random effect. Treating the mouse as a random effect basically assumes that the three mice have been randomly selected from an 'infinite' mouse population of that strain and its observed effect for a particular mouse is an observation of a random variable. Specifically, the F test statistic is:



where





where *ij *indexes the *i*th mouse for the *j*strain, and _*ij*_, _*j*_, and  are the means of normalized ratios for the *i*th mouse in the *j*th strain, all mice in *j*th strain, and over all strains, respectively. An F-value with degrees of freedom 4 and 10 for each gene is used to assess how variable the gene is among strains. An ANOVA table for this analysis is provided in Additional data file 2 (supplemental Table 1). To examine a possible dependence of statistical significance and signal intensity, we plotted the F-values versus the log_2_(intensity_Cy3 _+ intensity_Cy5_). There was no dependence on intensity for the significant genes either within strain or between strains (see Additional data file 2, supplemental Figure 2). The significance of these F-values was determined through estimating the pFDR, which is the proportion of falsely rejected hypotheses among the rejected hypotheses for pre-selected critical values [12]. As the overall goal here is to assess how genes vary among and within strains, it is natural to control the proportion of falsely rejected hypotheses among the rejected ones while examining the genes that vary among/within strains. In this paper, the pFDR level was set to be 0.10. This means that we expect 10% of our rejected hypotheses ('significant' genes) to have been falsely rejected. The pFDR level of 0.10 is a somewhat liberal cutoff as we are most interested in assessing overall levels of variation rather than defining a small subset of genes that vary with high confidence. A q-value that measures the strength of the F-value with respect to pFDR was also calculated for each gene using the algorithm proposed by Storey and Tibshirani [12]. The q-value is the minimum pFDR that occurs when rejecting a statistic with the observed F-value for the set of nested rejected regions. To avoid the distributional assumption, 1,000 bootstrap samples were used to calculate the pFDRs for a series of critical values and the q-values for all the genes.

To determine which gene ontology terms were enriched among the variable genes we used EASE software. EASE compares the proportion of genes that are assigned a given GO term among the list of variable genes to the proportion of genes with that GO term on the array as a whole. A statistical score similar to a *p *value is generated based on the upper bound of the distribution of Jackknife Fisher exact probabilities. For genes that varied within strain we performed separate EASE analyses for each strain, and then reported the GO terms that were enriched by >1.5-fold in at least 4 out of 5 strains and had the lowest average EASE score (cell growth, 0.35; amine metabolism, 0.25; cytokine activity, 0.39; ubiquitin ligase complex, 0.43).

Hierarchical clustering was performed using Cluster 3.0 software (Michael Eisen, Stanford University). We used complete linkage clustering for both genes and arrays with a correlation (uncentered) similarity metric with data either unweighted or weighted by F-value.

The normalized log ratios, F-values, q-values, and mean squares for the 2,382 genes assessed in the unpooled analysis are included in Additional data file 1. In addition, information about the microarray used in this study and the unprocessed gpr files may be obtained through the ArrayExpress website at the European Bioinformatics Institute [34]. The accession number is: A-MEXP-320.

### Quantitative RT-PCR

Quantitative PCR was performed using SYBR GREEN as a reporter as previously described [7]. Total RNA from each mouse liver was treated with DnaseI, purified using a Rneasy Minikit (Qiagen, Valencia, CA, USA), and 20 μg was used to generate cDNA for PCR reactions. Primers to ribosomal protein S16 were used to normalize for cDNA loading. The sequences of the primers used were: S16 forward, 5'-AGGAGCGATTTGCTGGTGTGGA-3'; S16 reverse, 5'-GCTACCAGGCCTTTGAGATGGA-3' (102 base-pair (bp) amplicon); Pfk2 forward, 5'-AAGAGGCCAAAGCTGGAGG-3'; Pfk2 reverse, 5'-GTCAGCATTCCGGTGGTGTA-3'; Cth forward, 5'-TCTTGCTGCCACCATTACGA-3'; Cth reverse, 5'-GCCTCCATACACTTCATCCAT-3'; Dnase2a forward, 5'-TCCAGGGAAAACTGCTGACC-3'; Dnase2a reverse, 5'-AGGAAAAGGCTGTCGGTGG-3'; Apoa4 forward, 5'-AGACAGGTGGTGGGGCAGGAC-3'; Apoa4 reverse, 5'-GCCCTCAGCCCATCACAGCAG-3'; Akr1e1 forward, 5'-CAAGGAGGGCGTGGTGAAGAG-3'; Akr1e1 reverse, 5'-GCTGGTGTGACTGGGTATGAC-3'; Cish forward, 5'-GGTGGGGCACAACATAGAGA-3'; Cish reverse, 5'-GGTGGCCAGACAGACAGGAG-3'; Socs2 forward, 5'-GGAATGGGACTGTTCACCTG-3'; Socs2 reverse, 5' GCAGAGTGGGTGCTGATGTA-3'.

## Additional data files

The following additional data are available with the online version of this paper. Additional data file 1 is a Microsoft Excel file containing the normalized log ratios, F-values, q-values, and mean squares for the 2,382 genes assessed in the unpooled analysis. Additional data file 2 contains two supplemental tables and three supplemental figures. Supplemental Table 1 shows the analysis of variance for the mixed effect model. Supplemental Table 2 shows selected genes with variable expression within mouse strains. Supplemental Figure 1, titled 'F-values are independent of intensity', shows plots of F-values versus intensity for each of the 2,383 genes analyzed both within and between strains. Supplemental Figure 2, titled 'Quantitative RT-PCR analysis on replicate mice confirms the expression variability patterns of ApoA4, Dnase2a and Socs2', shows transcript abundance measurements from independent RNA preparations from the same liver samples compared across different mice of different strains. Supplemental Figure 3, titled 'Comparisons of variances associated with array, mouse, and strain', shows the numbers of variable genes at specific average fold-changes across different mouse strains.

## Supplementary Material

Additional File 1 The normalized log ratios, F-values, q-values, and mean squares for the 2,382 genes assessed in the unpooled analysis Click here for file

Additional File 2 Supplemental Table 1 shows the analysis of variance for the mixed effect model. Supplemental Table 2 shows selected genes with variable expression within mouse strains. Supplemental Figure 1, titled 'F-values are independent of intensity', shows plots of F-values versus intensity for each of the 2,383 genes analyzed both within and between strains. Supplemental Figure 2, titled 'Quantitative RT-PCR analysis on replicate mice confirms the expression variability patterns of ApoA4, Dnase2a and Socs2', shows transcript abundance measurements from independent RNA preparations from the same liver samples compared across different mice of different strains. Supplemental Figure 3, titled 'Comparisons of variances associated with array, mouse, and strain', shows the numbers of variable genes at specific average fold-changes across different mouse strains. Click here for file

## Figures and Tables

**Table 1:** Variable genes within strains

	*129*	*Balb/c*	*CD1*	*FVB*	*CFW*
Total variable genes*	554(23%)	1,059(44%)	749(31%)	610(26%)	661(28%)
>1.5-fold difference	240(10%)	371(16%)	384(16%)	255(11%)	280(12%)
>2.0-fold difference	67(2.8%)	68(2.9%)	61(2.6%)	64(2.7%)	74(3.1%)
>3.0-fold difference	13(0.5%)	11(0.5%)	7(0.3%)	8(0.3%)	19(0.8%)

*At a pFDR of 10%
