# Supplementary material for: The contributions of normal variation and genetic background to mammalian gene expression
Source: Genome Biol. 2006 Mar 31;7(3):R26. doi: 10.1186/gb-2006-7-3-r26 (PMC1557746; doi:10.1186/gb-2006-7-3-r26)
Supplement: Additional File 2 — Supplemental Table 1 shows the analysis of variance for the mixed effect model. Supplemental Table 2 shows selected genes with variable expression within mouse strains. Supplemental Figure 1, titled 'F-values are independent of intensity', shows plots of F-values versus intensity for each of the 2,383 genes analyzed both within and between strains. Supplemental Figure 2, titled 'Quantitative RT-PCR analysis on replicate mice confirms the expression variability patterns of ApoA4, Dnase2a and Socs2', shows transcript abundance measurements from independent RNA preparations from the same liver samples compared across different mice of different strains. Supplemental Figure 3, titled 'Comparisons of variances associated with array, mouse, and strain', shows the numbers of variable genes at specific average fold-changes across different mouse strains. [file gb-2006-7-3-r26-S2.pdf]

Supplemental Table 1: Analysis of Variance for the Mixed Effect Model

Source of Variation	Deg. of Freedom	Mean Square	Expected Mean Sqaure
Dye	1	$30\sum_i(\overline{Y_{i...}}-\overline{Y_{....}})^2$	Q(dye) + σ^2
Strain	4	$12\sum_k(\overline{Y_{..k.}}-\overline{Y_{....}})^2/4$	$12\ \sigma^2_{strain}\ 4\ \sigma^2_{mouse} + \sigma^2$
Mouse	10	$4\sum_k\sum_j(\overline{Y_{.jk.}}-\overline{Y_{..k.}})^2/10$	$4\ \sigma^2_{mouse} + \sigma^2$
Error/Residuals	44	$\sum_i\sum_j\sum_k\sum_l(\overline{Y_{ijkl}}-\overline{Y_{ijk.}})^2/44$	σ^2

Notation: Let Y_{ijkl} be the log ratio for the *ijkl*th array, where *i* indexes the dye (i=1,2), *j* for mouse (j=1,2,3), *k* for the strain (k=1,2,3,4,5) and *l* for the replicate (l=1,2)

Supplemental Table 2: Selected genes with variable expression within mouse strains

GENE	HUGO	Accession	Fold Difference (q-value)				
			129	BAL	CD1	FVB	CFW
cytokine inducible SH2-containing	Cish	AI385595	1.3 (0.21)	7.9 (0.02)	3.1 (0.02)	3.1 (0.03)	2.3 (0.32)
suppressor of cytokine signaling 2	Socs2	AI464459	1.7 (0.03)	4.0 (0.02)	1.8 (0.02)	2.6 (0.03)	2.2 (0.03)
GDP-mannose pyrophosphorylase	Gmppb	AI323895	2.2 (0.03)	1.9 (0.02)	1.2 (0.02)	2.8 (0.03)	2.6 (0.15)
tetratricopeptide repeat domain 7	Ttc7	AI415173	2.0 (0.04)	2.0 (0.03)	2.3 (0.02)	2.3 (0.03)	1.7 (0.11)
RAB18, member RAS family	Rab18	NM_011225	4.7 (0.03)	1.8 (0.02)	1.3 (0.06)	1.3 (0.05)	1.2 (0.17)
lymphoid membrane protein	Lrmp	NM_008511	1.3 (0.08)	2.0 (0.02)	1.9 (0.03)	2.7 (0.03)	2.1 (0.03)
arrestin domain containing 3	Arrdc3	AI450344	2.2 (0.03)	2.1 (0.02)	1.8 (0.02)	2.1 (0.03)	1.6 (0.03)
transferrin receptor	Tfrc	AI426448	2.5 (0.03)	1.5 (0.02)	2.1 (0.00)	1.9 (0.03)	1.6 (0.03)
hematopoietic homeobox	Hhex	AI450826	2.4 (0.03)	2.5 (0.02)	2.1 (0.02)	1.3 (0.20)	1.2 (0.03)
vacuolar protein sorting 54	Vps54	AI452212	3.2 (0.03)	1.8 (0.02)	1.9 (0.04)	1.4 (0.15)	1.2 (0.06)
amiloride-sensitive cation channel	Accn3	AI414211	1.2 (0.09)	1.9 (0.02)	2.0 (0.02)	2.3 (0.03)	1.8 (0.03)
insulin-like growth factor 2 receptor	Igf2r	U04710	1.8 (0.11)	1.8 (0.04)	1.9 (0.08)	1.6 (0.05)	1.6 (0.03)
immunoglobulin superfamily 7	Igsf7	AI448699	1.4 (0.09)	1.5 (0.03)	1.7 (0.04)	2.1 (0.03)	1.8 (0.03)
delta sleep inducing	Dsip1	AI326808	1.4 (0.03)	2.7 (0.02)	1.5 (0.02)	1.6 (0.04)	1.4 (0.29)
bromodomain containing 2	Brd2	AI450617	2.7 (0.05)	1.8 (0.02)	1.7 (0.07)	1.2 (0.24)	1.2 (0.03)

Note: This table is a subset of genes that were variable at q>10% in at least 4 out of 5 strains. No ESTs or RIKEN genes were included. Genes expressed at a lower level in liver than in reference RNA were not included. Genes were selected by meeting the above criteria and sorted by average fold difference within strain.

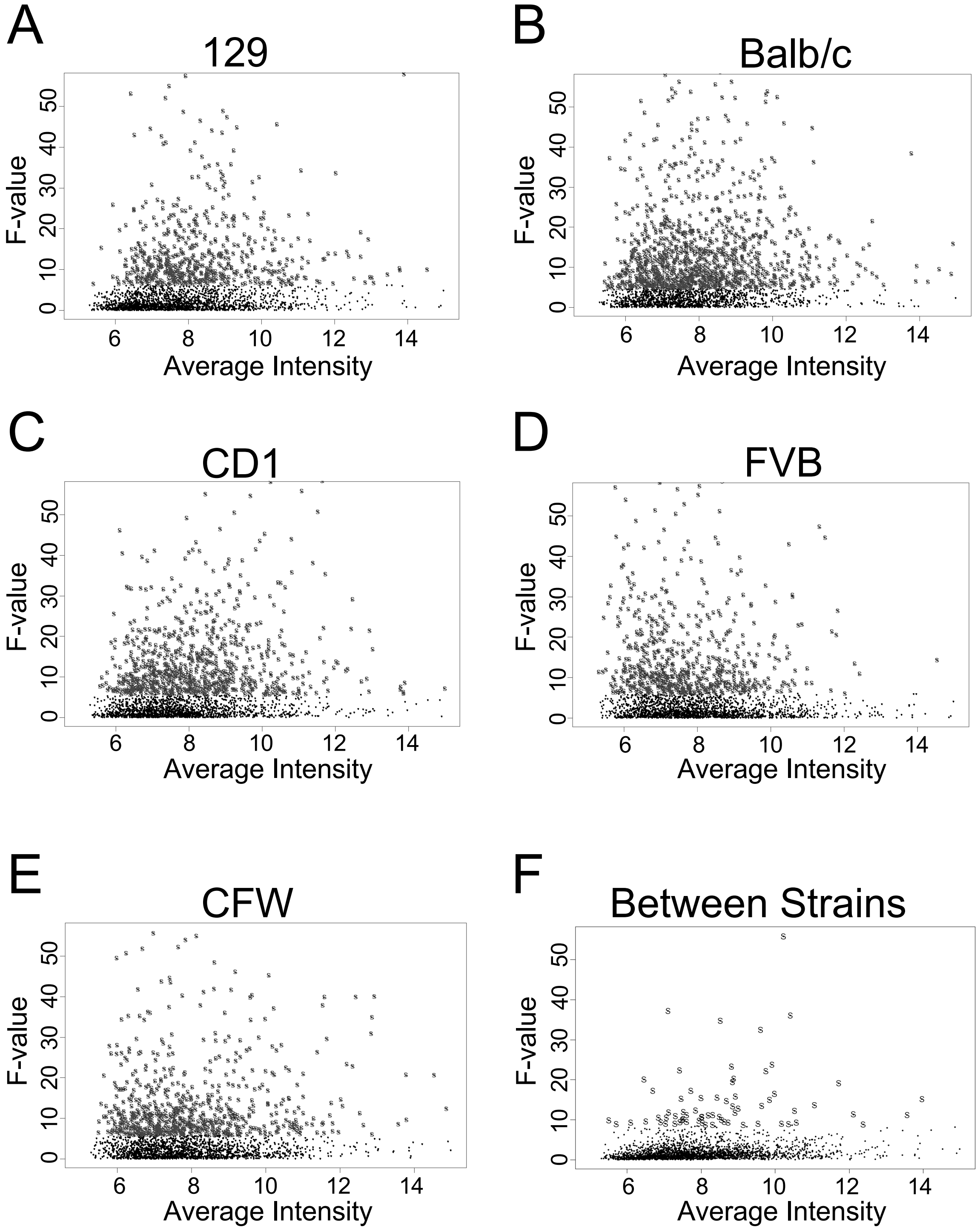

Supplemental Figure 1: F-values are independent of intensity.

F-values are plotted versus the average $\log_2(\text{intensity}_{Cy3} + \text{intensity}_{Cy5})$ for each of 2382 genes analyzed. Genes that were significant at pFDR of 10% are marked with an 'S'. Plots are shown for intra-strain analyses (A-E) and the inter-strain analysis (F). There is no obvious dependence of F-values on average intensity in either case.

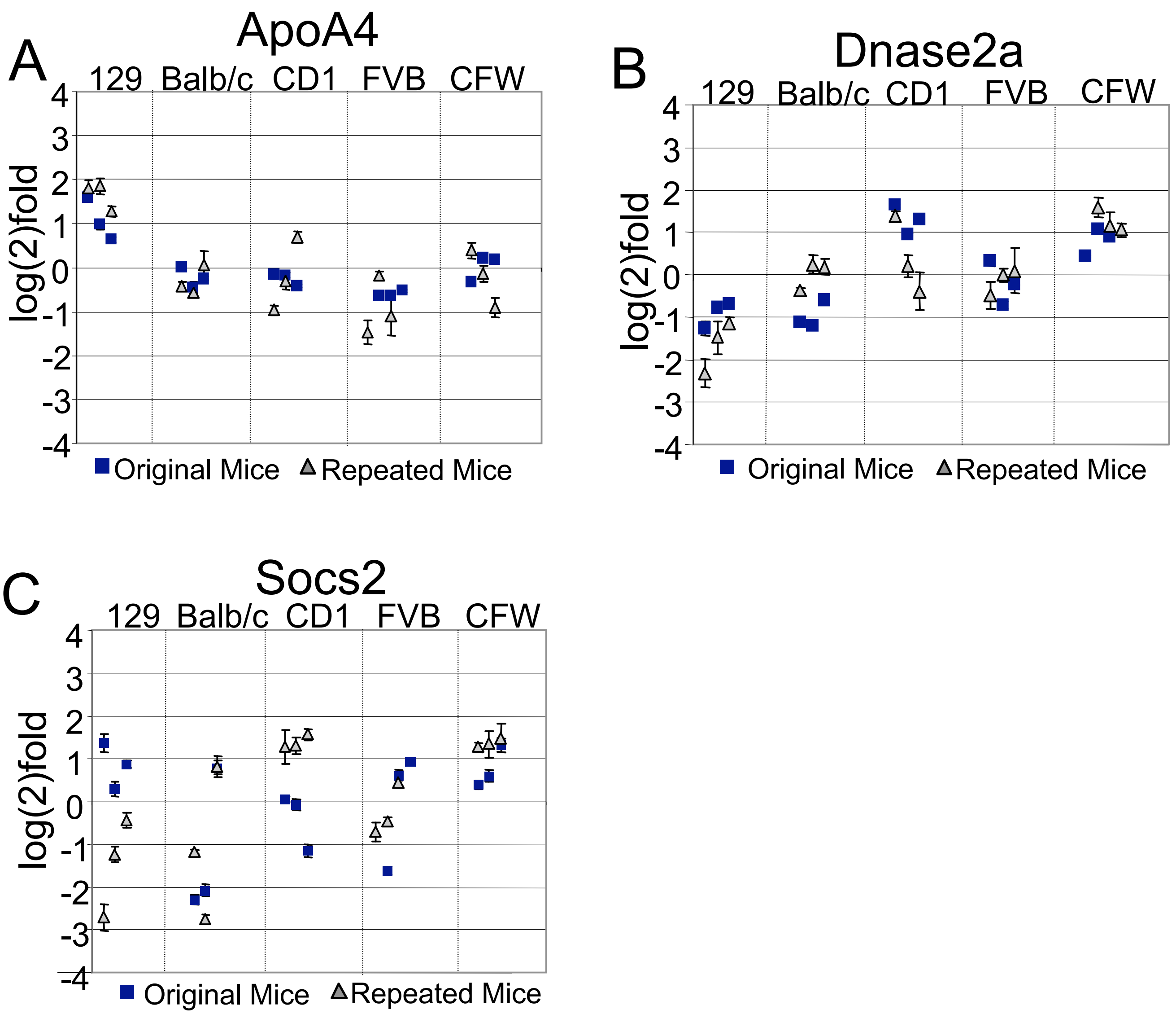

Supplemental Figure 2. Quantitative RT-PCR analysis on repeated mice confirms the expression variability patterns of *ApoA4*, *Dnase2a*, and *Socs2*. Quantitative RT-PCR measurements of *ApoA4*, *Dnase2a*, and *Socs2* transcript levels are depicted for both the original 3 mice used for the microarrays (blue squares) and three additional mice from each strain (gray triangles). Note that the data for *ApoA4* and *Dnase2a* from the original mice is the same as in Figure 1C, but presented in $\log_2$ format. In the repeated mice four independent RNA preparations were isolated from each liver (total of 12 RNA preparations per strain). Error bars represent the standard deviation of transcript measurements from the 4 RNA preparations, or from 4 replicate PCR reactions in the case of the original mice (note that for some measurements, the error bars fall within the square).

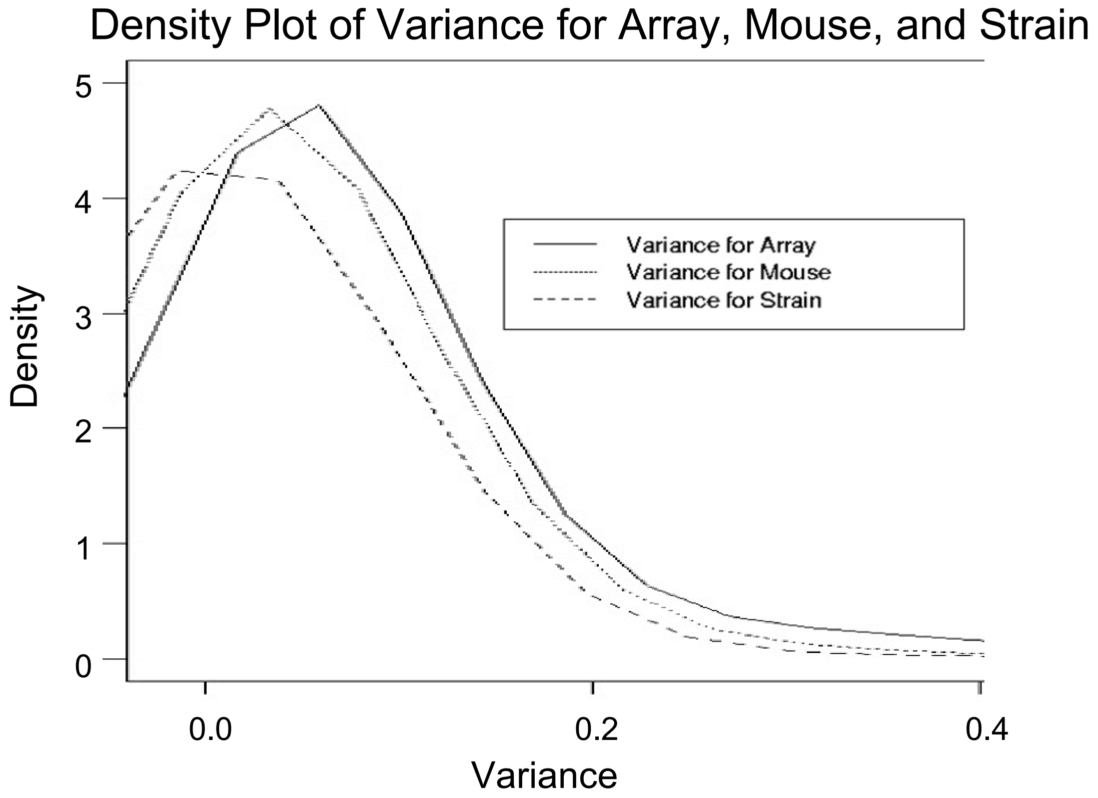

All 2382 Genes	129	Balb	CD1	FVB	CFW	Between Strains	Mean Array Fold Error
>1.5 fold	366	458	545	406	459	423	588
>2.0 fold	86	77	79	91	124	79	129
>3.0 fold	15	12	9	10	22	18	8

Supplemental Figure 3: Comparison of variances associated with array, mouse, and strain.

At a pFDR of 10% we found that 1876 genes (79%) varied within strain, while 66 genes varied between strains (2.8%), using a single test. A very small array variance (technical error) might result in more statistically significant genes within strain than between strains, even when variance due to strain is higher than the variance within strain. To address this we have drawn a density plot of the variances associated with array, mouse (within strain), and mouse (between strain) for all 2382 genes. The plot shows that the mouse (within strain) variances are higher overall than the mouse (between strain) variances, while the array variance is the largest overall. The total number of genes achieving at least 1.5 fold, 2 fold, or 3 fold differences is also tabulated for all genes; within strain, between strain, and for mean array fold error. The mean array fold error was calculated as the average fold difference between the maximum and minimum values of the 4 arrays for each mouse across all 15 mice. The overall mean array fold error is greater than mouse or strain fold differences, emphasizing that technical variation is not small relative to mouse (within strain) and mouse (between strain) variation. This analysis is difficult to formally interpret, but it supports the conclusion that variation specific to strain is a small component of overall inter-individual variation.
